# *Candida* and *Porphyromonas gingivalis*: the effect on wound closure *in vitro*

**DOI:** 10.1080/20002297.2017.1328266

**Published:** 2017-06-14

**Authors:** Thijs M. Haverman, Alexa M. G. A. Laheij, Johannes J. de Soet, Jan de Lange, Frederik R. Rozema

**Affiliations:** ^a^ Department of Oral Medicine, Academic Centre for Dentistry Amsterdam, University of Amsterdam and the Vrije Universiteit Amsterdam, Amsterdam, The Netherlands; ^b^ Department of Preventive Dentistry, Department Oral Medicine Academic Centre for Dentistry Amsterdam, University of Amsterdam and the Vrije Universiteit Amsterdam, Amsterdam, The Netherlands; ^c^ Department of Oral and Maxillofacial Surgery, Academic Medical Center Amsterdam, University of Amsterdam, Amsterdam, The Netherlands

**Keywords:** In vitro scratch assay, oral epithelial cells, re-epithelialization, oral ulceration, *Candida glabrata*, *Candida kefyr*, oral mucositis, wound closure, *Porphyromonas gingivalis*, cell migration

## Abstract

Microorganisms play a role in oral mucositis after cancer therapy. The current study explored the hypothesis that *Candida* spp. alone and together with Porphyromonas gingivalis cause delayed healing of oral ulcerations due to the inhibition of wound closure. An *in vitro* scratch assay model was used to study the influence of viable and heat-killed *Candida glabrata*, *Candida kefyr*, and *Candida albicans* on cell migration of oral epithelial cells. Separately, the effect of conditioned medium of *Candida* spp. and the effect of a mixed infection of *Candida* spp. with *P. gingivalis* on wound closure was studied. In the presence of 10 viable *C. glabrata* or *C. kefyr* versus one epithelial cell, with a multiplicity of infection (MOI) of 10, the relative closure of the scratch was 26% and 17%, respectively. At a MOI of 1, this was 60% for *C. glabrata* and 78% for *C. kefyr*. The inhibition of oral epithelial cell migration challenged with either *C. glabrata* or *C. kefyr* together with *P. gingivalis* was stronger than the inhibition caused by one of both organisms separately. *Candida* spp. inhibit cell migration *in vitro*. A combination of *Candida* spp. and *P. gingivalis* inhibited cell migration more than either microorganism separately.

## Introduction

One of the main functions of oral epithelial cells is to protect the underlying tissues from environmental influences in order to maintain homeostasis [1]. In case of oral ulcerations, the epithelial layer of cells is disturbed, and homeostasis is disrupted. Oral u lcerations are frequently encountered in patients with oral mucositis receiving cancer therapy [2]. Practically everyone undergoing radiotherapy for head and neck cancer, about 60–85% of hematopoietic stem-cell transplantation recipients, and 20–40% of patients receiving chemotherapy [3] develop oral mucositis. Clinically, oral mucositis can present in a variety of forms, ranging from erythematous lesions with intact mucosa causing food burn through to submucosal lesions that cause severe pain. Oral mucositis is associated with opioid use, dietary changes, parenteral feeding, weight loss, increased healthcare costs, and a higher risk of local and systemic infection [3–5].

The pathogenesis of cancer therapy–related oral mucositis used to be attributed to clonogenic cell death of basal epithelial cells. However, Sonis introduced a five-phase model to elucidate the pathobiology of oral mucositis, involving initiation, upregulation, and generation of messenger signals, signal amplification, ulceration, and healing [6]. The process is characterized by upregulation of pro-inflammatory cytokines, resulting in cell loss due to apoptosis and tissue necrosis, which leads to loss of mucosal integrity and bacterial translocation[7,8].

There is increasing evidence that microorganisms play a role in the pathogenesis of oral mucositis. In the five-phase model of Sonis, the role of oral microorganisms is undervalued. One of the functions of resident oral bacteria is the principle of colonization resistance to prevent colonization by exogenous and possible pathogenic bacteria [8]. It is suggested that cytotoxic therapy alters the ecological balance in the oral cavity, which causes a shift of the oral microbiome toward a more complex oral bacterial profile [8,9]. Wang et al. [7] hypothesized that cancer therapy leads to an ecological shift predominated by Gram-negative anaerobes, resulting in a dysbiotic ecosystem that initiates an inflammatory cascade. In a previous prospective clinical study, it was observed that the Gram-negative anaerobic *Porphyromonas gingivalis* was a positive predictor for the presence of oral ulcerations after hematopoietic stem-cell transplantation [10]. Thereafter, it was demonstrated that *P*. *gingivalis* strongly inhibits cell migration *in vitro* [11,12]. According to Stringer and Logan, the key signaling pathways associated with both host–microbial interaction and the development of mucositis include nuclear factor kappa B, Toll-like receptor signaling and mitogen-activated protein kinase signaling. They conclude that the altered oral microbiome has the potential to exacerbate the mucosal damage by potentiating apoptosis and the production of these pro-inflammatory cytokines [8].

In addition to bacteria, fungi may also be related to oral mucositis. In immunocompromised individuals, *Candida albicans* frequently overgrows the microbial flora and causes infections and epithelial damage [13,14]. *Candida* spp., particularly *C. albicans*, are associated with oral mucositis in patients with hematological malignancies [15,16]. In a study with head and neck cancer patients receiving radiotherapy, oral mucositis with pseudomembranous candidiasis was diagnosed in 77% (30/39) of the patients. *C. albicans* was the predominant species isolated, followed by *Candida glabrata* and *Candida kefyr* [14]. *C. glabrata* and *C. kefyr* were identified as positive predictors for mucosal ulcerations after hematopoietic stem-cell transplantation [10]. On the other hand, Westbrook et al. [17] and Epstein et al. [18] did not report a positive correlation between *Candida* colonization and the presence or severity of oral mucositis in stem-cell transplanted patients. The role of *Candida* spp. in the pathogenesis of oral mucositis remains to be elucidated in more detail [5].

The current study explored the effect of *Candida* spp. on epithelial cell migration *in vitro*. It as examined by using an *in vitro* scratch assay model, as previously described by Laheij et al. [12]. Epithelial cells were exposed to viable, heat-killed, and conditioned medium of *Candida* spp. From a previous study, it was already known that *P. gingivalis* is able to inhibit wound closure *in vitro* [12]. Therefore, the effect of a mixed infection of *Candida* spp. and *P. gingivalis* on epithelial cell migration was also studied. Finally, the effect of *Candida* spp. on oxygen saturation was studied, as it was hypothesized that the oxygen-reducing capacity of *Candida* spp. might be an important factor of interaction with *P. gingivalis*.

## Materials and methods

### Epithelial cells

The Japanese Collection of Research Bioresources (Osaka, Japan) provided the human buccal epithelial cell line H0-1-N-1. The cells were grown in an incubator at 37°C in a humidified atmosphere containing 5% CO_2_ and 95% air with Dulbecco’s modified Eagle’s medium Ham’s F-12 nutrient mixture (DMEM-F12; Invitrogen, Carlsbad, CA), completed with 10% fetal calf serum (Hyclone, Logan, UT), 100 IU/mL of penicillin, 100 μg/mL of streptomycin, and 250 ng/mL of amphotericin B (all from Sigma–Aldrich, St. Louis, MO) . After confluency was reached over the entire 75 cm^2^ bottle surface (Corning, New York, NY), the cells were detached using 0.25% trypsine-EDTA (Invitrogen) and counted with a hematocytometer. Finally, the cells were seeded in a 24-well plate in DMEM-F12 medium at a concentration of 3–5 × 10^5^ cells/mL.

### Bacterial strains and culture

*P. gingivalis* ATCC 33,277 was cultured anaerobically (80% N_2_, 10% H_2_, and 10% CO_2_) in Brain-Heart-Infusion (BHI; 37 g/L;BD Difco, Le Pont de Claix, France) supplemented with hemin (5 mg/L) and menadione (1 mg/L). *C. glabrata* CBS 138, *C. kefyr* CBS 1970, *C. albicans* BWP-17 (hyphal growth), *C. albicans* HGC-1 Δ/Δ (knockout with pseudo-hyphae), and *C. albicans* HGC-1 (complementation strain) were cultured aerobically at 37°C in amino acid–depleted, glucose-enriched Yeast Nitrogen Base (YNB; 6.7 g/L; BD Difco). The rationale for the mutant and the complementary strain was to study the influence of hyphae formation on cell migration. All microorganisms were grown until log phase, which was ascertained by measuring the optical density (OD). Subsequently, the microorganisms were washed twice with Dulbecco’s phosphate-buffered saline (DPBS; Invitrogen) and re-suspended in keratinocyte serum-free medium (SFM; Invitrogen) at a predetermined OD: *P. gingivalis* OD_690_ of 0.1 (corresponding to 5 × 10^8^ colony forming units [CFU]/mL) [12], *C. kefyr* OD_600_ of 0.7 (corresponding to 4 × 10^6^ CFU/mL), and OD_600_ of 1.0 for *C. glabrata* and *C. albicans* (corresponding, respectively, to 4 × 10^7^ and 3 × 10^7^ CFU/mL). The corresponding CFUs were determined beforehand. For each scratch assay, a freshly prepared bacterial and/or yeast culture was used. All cultures were checked for purity and hyphal growth by culturing and Gram staining.

### *Heat-killed* Candida

All *Candida* spp. were cultured and grown until log-phase and killed at 60°C for 60 min. The lack of viability was confirmed by plating cells on tryptic soy agar plates and incubating them at 37°C for 2 days. Gram staining was done to check for purity and hyphal growth and to confirm the presence of intact cell walls. After killing, the yeast cells were washed twice with DPBS and suspended in SFM at the required OD_600_ (the same as viable yeast cells) and frozen at −80^°^C until use.

### Conditioned medium

Again, all *Candida* spp. were cultured and grown until log-phase. After culturing, they were washed twice with DPBS, resuspended in SFM at the required OD_600_ (the same as viable yeast cells), and incubated aerobically for 5 h. After centrifuging, the yeast cells were removed. The conditioned medium was filter sterilized (0.2 μm; Sarstedt, Nümbrecht, Germany) and frozen at −80°C until use.

### Wound closure assay

The cells were seeded at a density of 3–5 × 10^5^ cells/mL in DMEM-F12 in 24-well plates and incubated until the cells reached confluency over the entire well surface. Before scratching a straight line in the monolayer of cells using a 1,000 μL blue pipette point (Greiner Bio-One, Alphen a/d Rijn, The Netherlands), the cells were washed once with DPBS. To remove any detached cells, they were washed twice with DPBS after scratching. In the experiments with viable (monoinfection) heat-killed, and conditioned medium of *Candida* spp., the wells were treated with 1 mL of solution (prepared as described above) with a corresponding multiplicity of infection (MOI) of 10 and 1. The wells exposed to a mixed infection were treated with 0.5 mL of solution of both organisms that was twice as concentrated. One milliliter corresponded to a MOI of 10 and 1 for *C. glabrata* and *C. kefyr* and a MOI of 100 and 10 for *P. gingivalis*. The control group was treated with SFM. Immediately and after 17 h, the scratch was photographed using an inverted digital phase contrast microscope EVOS FL (Advanced Microscopy Group, Mill Creek, WA). The relative migration distance was calculated with the formula percentage of closure of the treatment/percentage of closure of the control, in which the percentage of closure was calculated using the formula: 100 – [(scratch surface at 17 h/scratch surface at 0 h) × 100]. The surface of the scratch was calculated with Adobe Photoshop CS4 v11.0.1. The relative closure of the scratch under control conditions was 100%. Each treatment was performed in triplicate, and each experiment was performed on at least three separate occasions.

### Oxygen consumption

*C. glabrata* and *C. kefyr* were cultured in YNB and re-suspended in keratinocyte SFM. The oxygen consumption of both species was measured at an OD_600_ of 0.5. Oxygen consumption was measured using two AppliSens dissolved oxygen sensors coupled to a Bio Controller ADI 1030 (Applikon Biotechnology, Delft, The Netherlands). The electrodes measured the percentage of dissolved oxygen in the fluid. All the measurements were conducted under aerobic conditions in an incubator set at 37°C. The bijoux tubes were not sealed airtight so that the exchange of oxygen between the fluid and the air was still possible.

### Statistical analyses

Data of three or more separate experiments with similar conditions were assembled and analyzed. Friedman’s two-way analysis of variance was used to determine that the results of separate experiments had the same distribution of scores. Differences in relative closure of the scratch between different bacterial conditions were calculated with the nonparametric Mann–Whitney *U*-test. Statistical analysis was performed with IBM SPSS Statistics for Windows v23 (IBM Corp., Armonk, NY). A *p*-value of <0.05 was chosen as statistically significant.

## Results

### Influence of viable C. glabrata and C. kefyr on wound closure

**Figure 1.**
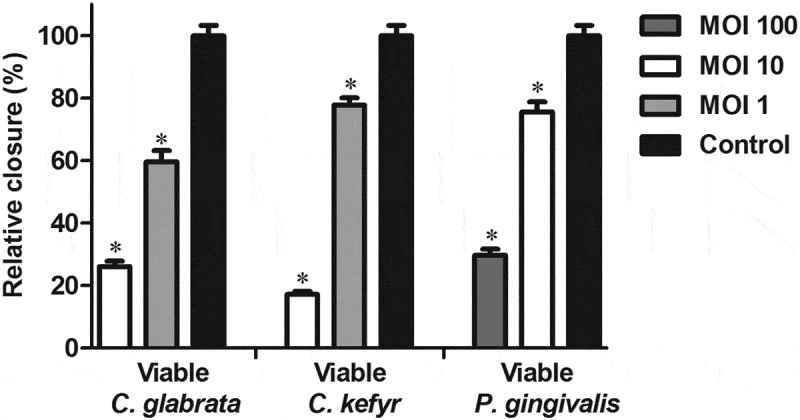
Relative closure (mean + standard error of the mean [SEM]) of scratched oral epithelial cells exposed to different concentrations of viable *Candida glabrata, Candida kefyr*, or *Porphyromonas gingivalis* compared to control. Difference with control is considered significant if *p* < 0.05 and is marked with an asterisk.

The relative closure of the scratch challenged with *C. glabrata* and *C. kefyr* compared to control is shown in Figure 1. In the presence of 10 viable *C. glabrata* versus one epithelial cell (MOI of 10), the relative closure of the scratch was 26%. At a MOI of 1, the relative closure of the scratch was 60%. For *C. kefyr* at a MOI of 10 and a MOI of 1, the relative closure was 17% and 78%, respectively. All conditions led to significantly more inhibition of closure of the scratch compared to control. Figure 1 also shows the relative closure of the scratch caused by *P. gingivalis*, as studied by Laheij et al. [12]. There was no significant difference in the inhibition of cell migration between *P. gingivalis* at a MOI of 10 and *C. kefyr* at a MOI of 1 (*p *= 0.518) and *P. gingivalis* at a MOI of 100 and *C. glabrata* at a MOI of 10 (*p *= 0.077). An assay in which the epithelial cells were exposed to viable *C. albicans* spp. could not be conducted due to thick biofilm formation of *C. albicans*, which made the scratch invisible.

### Influence of viable C. glabrata or C. kefyr with viable P. gingivalis on wound closure

**Figure 2.**
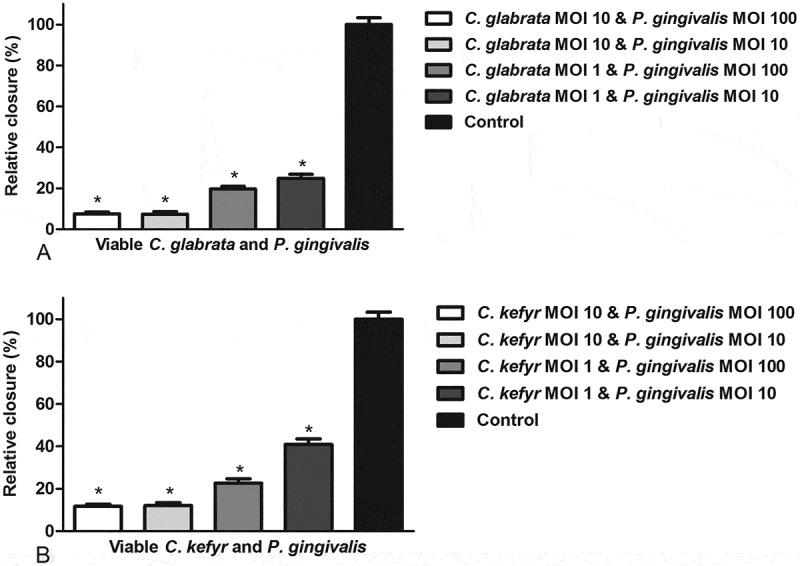
Relative closure (mean + SEM) of scratched oral epithelial cells exposed to different concentrations of viable *C. glabrata* (**A**) or *C. kefyr* (**B**) with *P. gingivalis* compared to control. Difference with control is considered significant if *p* < 0.05 and is marked with an asterisk.

**Figure 3.**
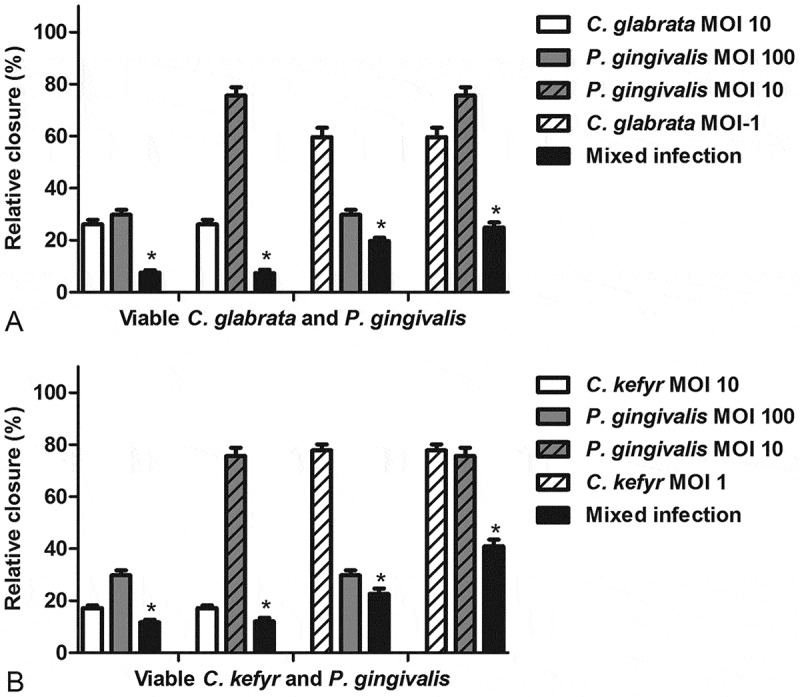
The difference (mean + SEM) between a mono and a mixed infection of *C. glabrata* (**A**) or *C. kefyr* (**B**) with *P. gingivalis*. Difference is considered significant if *p* < 0.05 and is marked with an asterisk.

The relative closure of the scratch exposed to a mixed infection of *C. glabrata* or *C. kefyr* and *P. gingivalis* is shown in Figure 2a and b. WIt confirmed viability of both *Candida* spp. and *P. gingivalis* on several different occasions by culturing at the end of the experiments (data not shown) All combinations of *C. glabrata* or *C. kefyr* and *P. gingivalis* led to significantly more inhibition of closure of the scratch compared to control. Moreover, the migratrion of epithelial cells was inhibited stronger when challenged with a mixed infection than challenged with one of the microorganisms separately. This was independent of the MOI (Figure 3a and b). Generally, a dose–response effect of the microbial concentration was observed (). However, when a concentration of a MOI of 10 of either *C. glabrata* or *C. kefyr* was present, an increase in concentration of *P. gingivalis* from a MOI of 10 to a MOI of 100 did not lead to more inhibition of closure of the scratch (). So when both *Candidas* were present at a MOI of 10, the presence rather than the concentration of *P. gingivalis* was responsible for the additional inhibitory effect of the mixed infection on cell migration.

### Influence of conditioned medium of and heat-killed C. glabrata, C. kefyr, and C. albicans on wound closure

The relative closure of the scratch in epithelial cells challenged with heat-killed *Candida* spp. did not differ from control. From conditioned medium only *C. albicans* BWP-17, a MOI of 10 did inhibit the relative closure of the scratch slightly, though significantly, compared to the control group (*x *= 88.9 vs. *x *= 100; *p *= 0.015).

### *Oxygen consumption of* C. glabrata *and* C. kefyr

**Figure 4.**
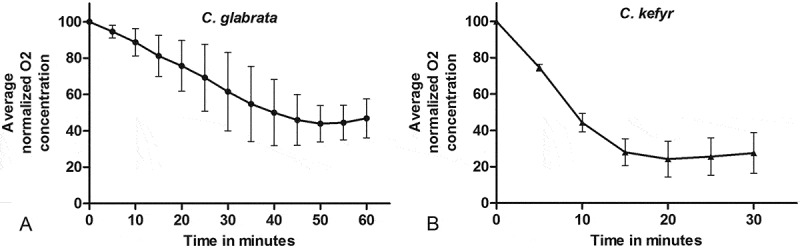
Average normalized oxygen (O_2_) concentration in serum-free medium with *C. glabrata* (**A**) or *C. kefyr* (**B**).

The graphs in Figure 4a and b display the average normalized oxygen concentration in SFM. Both *C. glabrata* and *C. kefyr* caused a clear decrease in oxygen over a relatively short period of time. The relative decrease in oxygen by *C. kefyr* was greater and more rapid compared to *C. glabrata*.

## Conclusion

In our previous clinical study, *P. gingivalis* in particular, and at the same time *C. glabrata* and *C. kefyr*, were identified as explanatory variables of oral ulcerative mucositis in patients undergoing hematopoietic stem-cell transplantation [10]. In an *in vitro* scratch assay, a model for wound healing, it was found that *P. gingivalis* inhibited epithelial cell migration, thereby possibly delaying the healing of oral ulcerations *in vivo* [12]. The current study found that *Candida* spp. were able to inhibit cell migration too when using the same *in vitro* scratch assay . To the authors’ knowledge, this is the first study to report an inhibitory effect of *C. glabrata* and *C. kefyr* on the migration of oral epithelial cells *in vitro*. Unfortunately, it was not possible to study the effect of viable *C. albicans* on cell migration due to thick biofilm formation.

Interestingly, in this study, heat-killed *C. glabrata, C. kefyr*, and *C. albicans* and conditioned medium from these species did not inhibit cell migration. From this, it can be concluded that the inhibitory effect of these *Candida* spp. is dependent on a certain minimal cell-bound enzymatic or metabolic activity and is associated with temperature sensitive cell-wall proteins. The inhibitory effect is not associated with secreted metabolites and signaling molecules, or with temperature unsensitive cell-wall proteins. This is in contrast to conditioned medium of *P. gingivalis* and heat-killed *P. gingivalis*, which did inhibit epithelial cell migration [12].

The effect of a mixed infection of *Candida* spp. and *P. gingivalis* on the inhibition of epithelial cell migration was also studied. The inhibition of cell migration challenged with a mixed infection was stronger than the inhibition caused by one of both microorganisms separately. The inhibiting effect might partly be attributable to the oxygen-reducing effect of both *Candida* spp. Within a biofilm, bacterium–fungus interactions influence the overall survival and proliferation of the respective species [19]. *C. albicans* promotes growth and biofilm formation of anaerobic bacteria under aerobic conditions [20,21]. An explanation for this might be that *Candida* creates a pro *P. gingivalis* anaerobic microenvironment by using oxygen for its own metabolic processes (metabolic interaction). A fast and large reduction of oygen was observed in SFM medium if *C. glabrata* or *C. kefyr* was present. Moreover, the lower oxygen levels might influence the viability of epithelial cells. Hieke et al. found that under anoxic conditions, the cell count of gingival epithelial cells was reduced to 75% after 24 h, 60% after 48 h, and 30% after 72 h compared to aerobically cultivated cells. Moreover, they noticed that the metabolic activity of the epithelial cells was reduced. However, the influence of oxygen levels on cell viability was not subject of the present study [22].

Epithelial cell death was not excluded as a mechanism of inhibition of epithelial cell migration in this study. However, during all experiments, the epithelial cells were strongly attached to the surface, and the cells looked morphologically viable. Moreover, in a previous study, using the same model, epithelial cell viability was confirmed [12]. Therefore, epithelial cell death would not appear to be the mechanism that is responsible for the inhibition of cell migration by *Candida* spp. that was observed in this study.

The present study found that the presence rather than the concentration of *P. gingivalis* was important for the additional inhibitory effect on cell migration when both *Candida* spp. were present at a MOI of 10. First, it is possible that the inhibitory effect on epithelial cell migration of *Candida* and *P. gingivalis* is at its maximum at a MOI of 10 within the model that was used. Another explanation might be that one *Candida* cell can only interact with a certain amount of *P. gingivalis*, which means that after a certain threshold, extra *P. gingivalis* does not result in an additional effect. In the current study, *C. glabrata* and *C. kefyr* were found to be inhibitors of wound closure. This study supported the previously reported clinical data, in which *Candida* spp. were identified as positive predictors of oral ulcerative mucositis in hematopoietic stem-cell transplant recipients [10]. Both findings support the idea that *Candida* spp. might play a role in the complex process of ulcerative oral mucositis.

In conclusion, *C. glabrata* and *C. kefyr* strongly inhibited the migration of oral epithelial cells in an *in vitro* scratch assay. Moreover, a combination of *Candida* spp. and *P. gingivalis* inhibited cell migration more than either microorganism separately. In this interaction, *Candida* might play an essential role by creating a pro *P. gingivalis* anaerobic microenvironment. Heat-killed *C. glabrata, C. kefyr*, and *C. albicans* and conditioned medium from these species did not inhibit cell migration.
